# Untargeted metabolomics reveals the effect of lovastatin on steroid-induced necrosis of the femoral head in rabbits

**DOI:** 10.1186/s13018-020-02026-5

**Published:** 2020-10-28

**Authors:** Xiangnan Ren, Zixing Shao, Wu Fan, Zixuan Wang, Kaiyun Chen, Xuefeng Yu

**Affiliations:** 1 Key Laboratory of Bioorganic Phosphorus Chemistry and Chemical Biology (Ministry of Education), Department of Chemistry, Tsinghua University, Beijing, 100084 China; 2 The Fourth Affiliated Hospital, Nanchang University, Nanchang, 330003 China; 3 Beijing Institute of Nutritional Resources, Beijing, 100069 China; 4 Nanchang University, Nanchang, 330006 China

**Keywords:** Lovastatin, Metabolomics, Femoral head necrosis, Steroid-induced

## Abstract

**Purpose:**

Lovastatin is an important medicine and it shows a significant effect against glucocorticoid-induced necrosis of the femoral head. This study aimed to investigate the effect of lovastatin on preventing necrosis of the femoral head of by serum metabolomics strategy.

**Methods:**

Adult healthy adult Japanese white rabbits were divided into three groups: control group, model group, and drug group. The pathologic changes of femoral head were assessed with magnetic resonance imaging and microscope. Metabolomics based on ultra-high performance liquid chromatography tandem mass spectrometry analysis was used to analyze the collected serum sample. Data were analyzed using principal component analysis, partial least squares-discriminate analysis, and orthogonal partial least squares-discriminant analysis. All potential metabolites were identified by comparing with human metabolome database, Metlin database, lipid maps, and chemspider database.

**Results:**

Eleven potential biomarkers were noted and identified as potential biomarkers. The change of biomarkers suggested that lovastatin on preventing necrosis of the femoral head may affect glycerophospholipid metabolism, linoleic acid metabolism, sphingolipid metabolism, alpha-linolenic acid metabolism, pyrimidine metabolism, and arachidonic acid metabolism.

**Conclusion:**

The study suggested that lovastatin could prevent the glucocorticoid-induced necrosis of the femoral head of rabbits. The possible reasons were closely associated with adjusting the lipid metabolism, inhibiting adipogenesis, and delaying the osteocyte apoptosis.

**Supplementary information:**

**Supplementary information** accompanies this paper at 10.1186/s13018-020-02026-5.

## Article summary

### Article focus

This study aimed to investigate the effect of lovastatin on preventing necrosis of the femoral head of by serum metabolomics strategy.

### Key messages

Metabolomics based on ultra-high performance liquid chromatography tandem mass spectrometry analysis (UHPLC-MS/MS) was used to analyze the collected serum sample. Data were analyzed using principal component analysis, partial least squares-discriminate analysis, and orthogonal partial least squares-discriminant analysis.

### Strengths and limitations

#### Strengths

Metabolomics study based on UHPLC-MS/MS was applied to investigate the serum metabolite profiling of rabbits of steroid-induced necrosis of the femoral head. Potential biomarkers related to lovastatin for treatment of steroid-induced necrosis of the femoral head were discovered and verified, and their metabolic pathways were discussed. Nuclear magnetism imaging and observation on histopathology were carried out.

#### Limitations

Firstly, the serum samples were collected from rabbits, which could provide the information of treatment for the use of lovastatin on steroid-induced avascular necrosis of the femoral head (SANFH). The samples from clinical patients needed further study. Moreover, the kinds of statins were not considered and we focused on the effect of lovastatin on SANFH. Additionally, this study gave the possible reason why lovastatin could prevent the SANFH. But the precise mechanism still requires a further in vivo study.

## Introduction

Glucocorticoid-induced necrosis of the femoral head is difficult miscellaneous diseases in clinical diagnose [1]. The glucocorticoid is one of the steroid and plays an important role on clinical treatment of organ grafting, systemic lupus erythematosus, rheumatic arthritis, dermatomyositis, and myasthenia, but long time or excess use of the glucocorticoid could result in escalation of osteonecrosis [2–5]. Researchers have paid more attention to steroid-associated osteonecrosis in the recently years. The effect of glucocorticoid for disease treatment is remarkable. However, because of the side effect, it is restrictive for using of glucocorticoid, resulting in influencing the treatment of diseases which need chronic or excessive use glucocorticoid [6, 7]. Therefore, the effective treatment of steroid-induced avascular necrosis of the femoral head (SANFH) was appealing.

The hypotheses for the pathogenesis of SANFH were complex and comprehensive, including lipid metabolism disorder, intravascular coagulation, microvascular injury, intraosseous hypertension, osteocyte apoptosis, osteoporosis, enlarged fat cells and fat embolism, and so on [2–4, 8, 9]. The lipid metabolism disorder was nuclear factor. Firstly, glucocorticoids could lead to gather the blood cells within the capillaries and increase the content of blood lipid and plasma protein, resulting in tissue ischemia or hypoxia. Secondly, glucocorticoids could promote fat decomposition, inhibit fat synthesis and the use of glucose by peripheral tissue, increase cholesterol and triglyceride levels, and lead to hyperlipidemia. The hyperlipidemia may lead to the formation of fat embolus in the femoral arteries and cause ischemia or necrosis. Thirdly, glucocorticoids could promote a large number of bone marrow stromal cells to transform the adipocytes, resulting in intramedullary adipose tissue accumulation, coagulation disorders, and elevation of intracranial pressure. Fourthly, the accumulation of fat in liver could release fat embolism, which attached to the damaged blood vessel wall and caused microcirculation disorder [10–13]. The drug therapies for SANFH included the statins, anticoagulant, and anti-osteoporotic drugs. Some anticoagulant and anti-osteoporotic drugs were reported the effect of prevention and cure for SANFH [14]. Researchers discovered that the statins could prevent the SANFH through correcting the lipid metabolism disorder [15, 16]. Yin H et al. [15] believed that simvastatin could enhance the effects of multiple decompressions in preventing progression of SANFH and reducing the risk of femoral head collapse. Nozaki Y et al. [16] found that pravastatin may be effective in reducing steroid-induced osteonecrosis of the femoral head. The statins were important drug for treatment of SANFH and the possible theory was lipid-lowering therapy. However, it is unclear what kind of action path and how improve the femoral head necrosis? Hence, the mechanism of statins on steroid-induced necrosis of the femoral head needs to be studied clearly.

Rats and rabbits are often used to construct the femur head necrosis model. Due to the high mortality rate of hormone-induced femur head necrosis model, researchers have selected rabbits to construct the femur head necrosis model and used in the lovastatin intervention SANFH study for many years. In the experiment, the rabbit weight (> 2.5kg), feeding environment, antibiotic use, and other factors were strictly controlled, which greatly improved the success rate of modeling and ensured the smooth completion of the experiment [17].

Metabolomics involves the researchers of entire pattern of small molecular weight compounds of endogenous or exogenous metabolites in biological samples [18]. Recent reports have suggested that metabolomics by liquid chromatography tandem mass spectrometry (LC-MS) could research the disease mechanism and drug effect [19–26]. Especially for untargeted metabolite analysis, ultra-high performance liquid chromatography tandem mass spectrometry analysis (UHPLC-MS/MS) could obtain the comprehensive metabolic changes, which contribute to evaluate the effect of drug and explore the mechanism [24, 27–34]. However, metabolomics study on the effect of statins for treatment of steroid-induced necrosis of the femoral head has not been reported.

In this study, metabolomics study based on UHPLC-MS/MS was applied to explore the serum metabolite changes of rabbits of SANFH administrated by lovastatin, which was one of the statins. Potential key biomarkers associated with lovastatin for treatment of SANFH were discovered and verified, and their metabolic pathways were discussed. Furthermore, nuclear magnetism imaging and histological examination were carried out for the phenotype proof of lovastatin effect for SANFH.

## Materials and methods

### Chemical and reagents

Sodium pentobarbital solution, normal saline, and lovastatin was obtained from the Fourth Affiliated Hospital of Nanchang University. Organic reagents, such as acetonitrile, methanol, isopropanol, were HPLC grade and purchased from Fisher (USA). Prednisolone acetate injection was purchased from the company of Xianju pharmacy (Zhejiang, China). Ultrapure water was purified by a Milli-Q system (Millipore, Bedford, MA, USA).

### Animal handling

Rabbit care and use were conducted in accordance with the recommendations in the Guide for the Care and Use of Laboratory Animals of the National Institutes of Health. A total of 70 healthy adult Japanese white rabbits were purchased from Animal Center of Tsinghua University. After 1 week, the rabbits were gotten venous blood to analyze the biochemical index. Except the biochemical index anomaly and the accidental death, 70 healthy adult Japanese white rabbits were randomly divided into three groups, namely control group, model group, and drug group. Sixteen rabbits for control group, 27 rabbits for model group, and drug group. Model group and drug group were injected prednisone solution (10 mg/kg) while control group was injected the same volume normal saline into left hip muscle, twice a week for 4 weeks. Drug group was administered intragastrically with lovastatin water solution every other day at a dose of 10 mg/5 mL per time throughout the whole experiment for 6 weeks. Control group and model group were administered intragastrically with the same dosages warm distilled water. Except the accidental death and the killed rabbits, the residue rabbits were gotten venous blood 1 week, 2 weeks, 3 weeks, 4 weeks, 5 weeks, and 6 weeks later. The minimum sample size of metabolomic at each weekly time point was 6.

#### The diagnostic criteria of SANFH

The rabbit model has dull color, listlessness, and lameness. We can see some histopathological changes that the femoral head trabeculae are thin, broken, and irregularly arranged; bone lacunae collapse and bone nuclei contract, dissolve, or disappear; and there are abundant adipocytes and eosinophils with peripheral bone marrow necrosis under a light microscope. Magnetic resonance imaging (MRI) scan on weighted images of femoral head showed annular and banded uneven low-signal areas and high-signal joint cavity effusion. There was no obvious change in the shape and contour of femoral head and articular cartilage.

### Sample collection and preparation

According to the related reference [35], the samples were taken at the specific nodes and serum samples were prepared. Above 3 mL, venous blood was gotten from specific rabbits. The blood samples were centrifuged for 10 min (4 °C, 3500 r/min) to collect the serum sample. On the analyzing day, after serum sample thawed, vortex 3 min by using Vortex Mixer XW-80A. Then, 100 μL serum sample was taken for metabolomics analysis. The proteins in sample were removed by using 400 μL acetonitrile. The supernatant was evaporated to dryness by Centrivap Concentrator (LABCONCO, USA). The residue was reconstituted in the initial mobile phase, vortex 3 min, and ultrasonic extracted 5 min at 4 °C. Through the centrifugation, supernatant was transferred into 150 μL glass insert in a 1.5 mL amber glass vial and analyzed.

### Magnetic resonance imaging scan

The rabbits were fastened and injected by 35 mg/kg 1 % sodium pentobarbital solution. The coronal scan of bilateral femoral heads of all rabbits was performed with Philips Achieva 3.0T TX MRI. The indexes were as follows: 3D T_2_WI VISTA (TR 2600 ms, TE 200 ms), slice thickness 1.2 mm, flip angle 90°, and matrix (reconstruction 256 × matrix scan 256). Then the MRI results of lovastatin for treatment of SANFH were researched [35].

### Histological Examination

The femoral heads of rabbits in the experimental groups were taken at 0 week and 6 weeks after experiment process. The femoral heads were prepared by washing, decalcification, paraffin embedding, and HE staining [3]. After the plasma sample collection and MRI scan of the femoral head, the rabbit model was fixed. After ether anesthesia, one side of the femoral head bone tissue was fixed with 10 mg decalcification. Preparation of paraffin sections: washed the slides and soaked them in acid overnight. Rinsed the slides with running water for 3 times and rinse them with distilled water for 3 times. Paraffin embedding: after fixation, the specimens were fully decalcified with 15%EDTA decalcification solution. After decalcification, rinse EDTA repeatedly with 1‰ DEPC water. After dehydration, transparent with xylene, soak for 2 h and 2 times. After being taken out, immersed in paraffin for 10 min (constant temperature 60 °C) and replaced the paraffin for 2 h. After the paraffin wax solidifies, removed the wax block. Making of slices: the tissue was sectioned with a rotary tissue slicer (the thickness of the sections was 5 μm). After being placed in distilled water, the slides were picked up and baked overnight in a 60 °C thermostat. Paraffin sections were prepared. HE staining method: paraffin-embedded specimens were consecutively sectioned (5 μm) and stained. Conventional optical mirror specimens were prepared by sealing with optical gum. The signal acquisition and analysis system from optical microscope (DSX100, Olympus, Japan) was used for observation, analysis, and assessment.

### UHPLC-MS/MS analysis

UHPLC-MS/MS analysis was performed using Q Exactive Plus High Resolution Mass Spectrometer (THERMO, USA) equipped with Ultimate 3000 (DIONEX, THERMO, USA). Acquity™ UPLC BEH C18 column (2.1 × 100 mm, 1.7 μm) (Waters, USA) was used. The chromatographic condition in reference was used [35]. The mobile phase consisting of isopropanol/facetonitrile 50:50 (V/V) with 0.1% formic acid (mobile phase A) and 0.1% formic acid solution (mobile phase B). The gradient elution process was 0–2 min, 2% A; 2–22 min, 2–99% A; 22–25 min, 99% A; 25.1–30 min, 2% A. The injection volume was 10 μL. The mass spectrometer was operating in full MS mode at a mass range of 100–1500 m/z in positive ionization.

### Statistical analysis and metabolite identification

The raw data were acquired using Xcalibur Qual Browser (THERMO, USA). The data were imported into the Progenesis QI (Waters, USA) for data analysis. Pre-treatment procedures, such as peak finding, alignment, filtering, and normalization, were performed to process the raw data. Principal component analysis (PCA) was used to obtain an overview of variations among the different groups. For the multivariate analysis, partial least squares-discriminate analysis (PLS-DA) and orthogonal partial least squares-discriminant analysis (OPLS-DA) were conducted with EZinfo 3.0 software. For LC/MS datasets, peaks were preliminarily identified by referring to online databases (HMDB, METLIN, Basic lipid, Glycerol lipid and Lipid MAPS). The results were matched with the molecular and fragment ions of experiment MS/MS spectra as well as confirmed with that of literatures [30, 31].

The Heml 1.0.3.7 software packages were used to make a heat map to show the relative amount of differential metabolites and to give their hierarchical cluster analysis (HCA) [35, 36]. The pathway analysis of the dataset of identified key metabolites was also performed using MetaboAnalyst [35]. The identified key metabolites at different time of trial groups were shown in the histogram and were proceeded normality test and one-way ANOVA by SPSS software (Ver. 19.0) (IBM, USA). Differences were considered significant at *P* < 0.05.

### Quality control

For validating the stability of the analysis process, quality control sample (QC) was first prepared by mixing serum from each sample (10 μL) [35]. QC was injected ten times before the batch process during the analysis process, to monitor the stability of sample preparation and instrument.

## Results

**Fig. 1:**
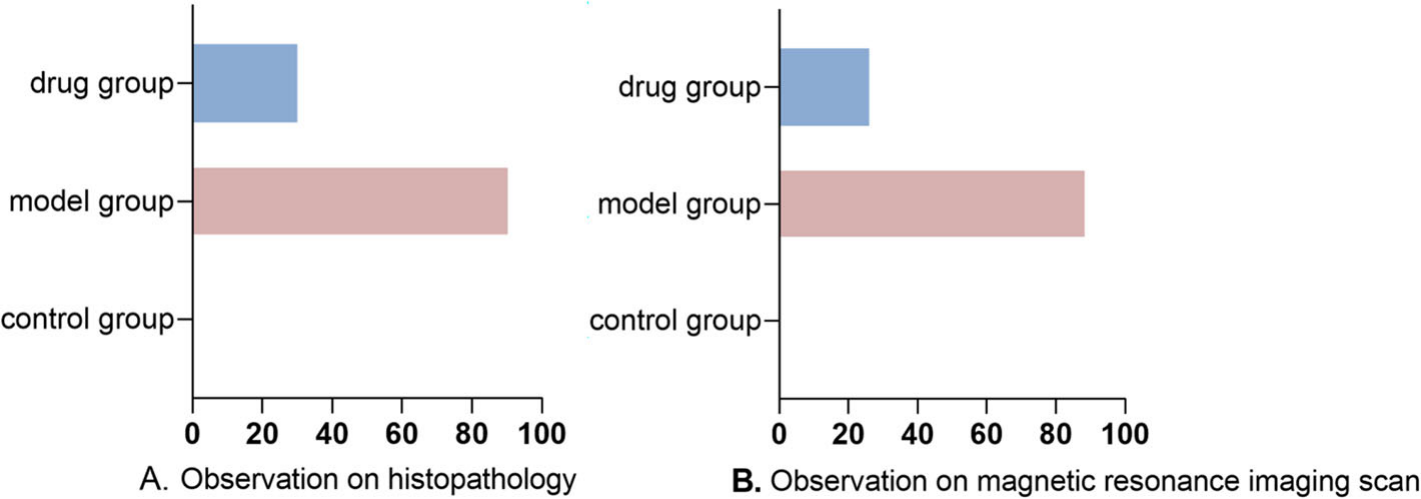
The incidence of osteonecrosis in each group

According to the imaging observations, the rate of osteonecrosis in the model group was above 90%, which met its construction standard. And the rate of osteonecrosis was reduced by 60–70% in the drug group (Fig. 1).

### Multivariate data analysis

**Fig. 2:**
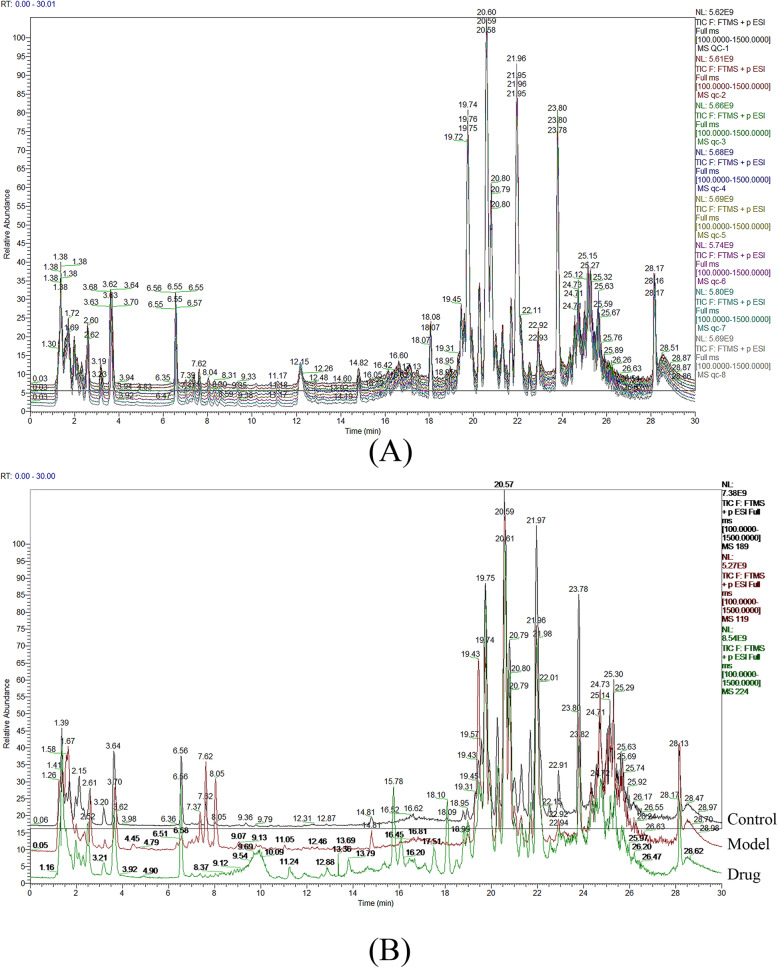
Typical LC-MS total ion chromatograms (TIC) from serum samples of the quality control (QC) group (**a**) and other groups (**b**) (black: control sample, red: model sample, green: drug sample)

**Fig. 3:**
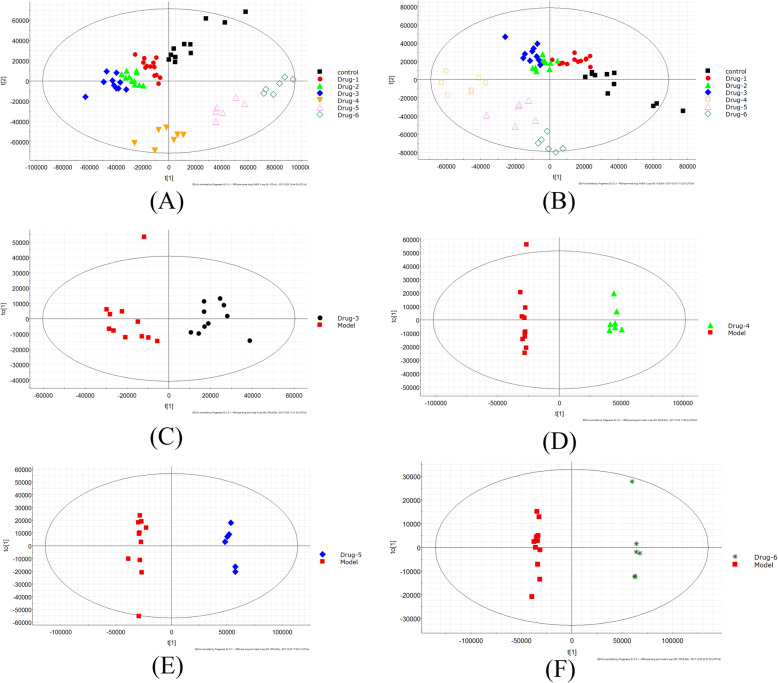
The score plots following **a** PCA, **b** PLS-DA among the groups, and **c**–**f** OPLS-DA analysis between model group and drug group (3 weeks, 4 weeks, 5 weeks, and 6 weeks)

There were a total 14 rabbits that suffered from the accidental death. At each weekly time point, 3 rabbits were examined by magnetic resonance imaging scan, then were killed and taken to histological examination. A pooled quality control (QC) sample was repeatedly analyzed during sample runs to evaluate the analytical stability of LC-MS-based methods for global serum metabolic profiling. The overlapped total ion current (TIC) chromatograms of the QC sample demonstrated the strong repeatability of our LC-MS system (Fig. 2a). Typical TIC chromatograms of the serum metabolic profiles of the control sample, model sample, and drug sample analyzed by LC-MS were shown in Fig. 2b. Obvious separation trend between control sample and the different time of drug sample was observed in principal component analysis (PCA) score plot (Fig. 3a). Subsequently, partial least squares-discriminate analysis (PLS-DA) was applied to the classification of control sample and the different time of drug samples (Fig. 3b). The trend of different time points after drug administration gave an intuitive result to evaluate the therapeutic effect. After 1 week, 2 weeks, and 3 weeks administration of drug, the serum sample points of rabbits were close to each other, despite being separated. However, groups of 4 weeks, 5 weeks, and 6 weeks after administration of drug could be clearly separated since the endogenous metabolites changed remarkably in these three groups. Although the PCA and PLS-DA figure provided an overview of the groups, the variables responsible for differences in each cluster were still unclear. The orthogonal partial least squares-discriminant analysis (OPLS-DA) as a supervised pattern recognition approach was used to find the metabolites. Figure 3c–f showed the loadings of serum different phases when model group compared with different time of drug group. Supplementary Fig. 1 showed the S-plots of serum different phases and the goodness of fit. In OPLS-DA model, the values of *R*^2^*Y* and *Q*^2^ were close to 1.0, which suggested that models established in this study had good fitness and prediction.

### Metabolites identification

**Fig. 4:**
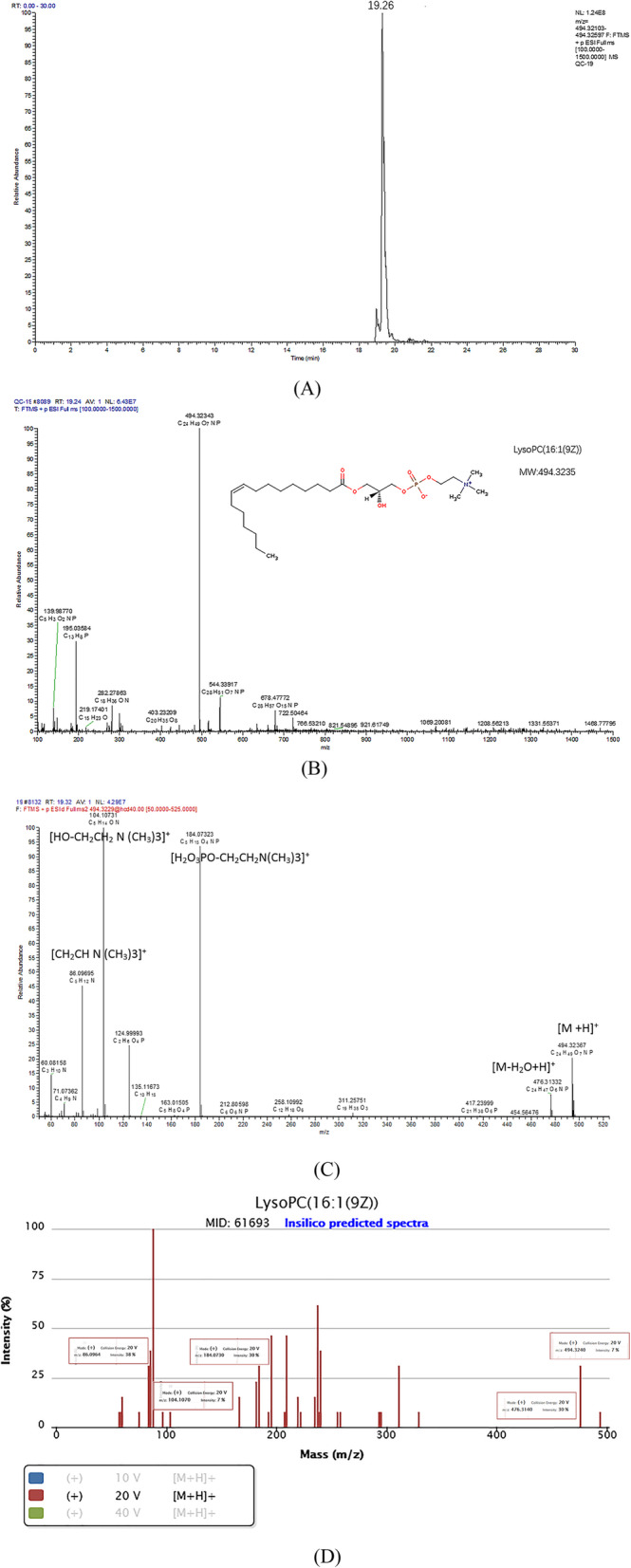
Identification of a selected marker. **a** Peak of potential biomarker of m/z 494 in extracted ion chromatogram with positive mode. **b** Compound structure and corresponding mass spectrum. **c** MS/MS spectrum on the collision energy was 20 eV. **d** standard MS/MS fragmentation in Metlin Databases

According to the tag of S-plots and *P* < 0.05, CV < 0.3, 22 representative metabolites were detected between drug group and model group. In our study, biomarkers were tentatively identified based on accurate mass measurements via UHPLC-MS/MS, comparison with theoretical to observed isotopic patterns, MS/MS fragment or comparison with HMDB database (http://www.hmdb.ca/spectra/ms/search), Metlin database (https://metlin.scripps.edu), lipid maps (http://www.lipidmaps.org/), and chemspider database (http://www.chemspider.com/). To illustrate the potential biomarker identification process, one metabolite (*t*_R_ = 19.26 min, m/z 494.3235) was detailed as an example to be described. First of all, extracted ion m/z 494.3235 in chromatogram, the retention time 19.26 was obtained and was the same as measurement (Fig. 4a). Additionally, the assistant software packed in Xcalibur was used to determine the elementary composition for the peak at m/z 494.3234. The calculated list provided several possible element compositions when considering the elements of carbon, hydrogen, nitrogen, oxygen, and phosphorous (C, H, N, O, and P). The lower the error value, the better the fit. After a series of analysis, only one possible element composition of C_24_H_49_NO_7_P was obtained and it was the ion of [M + H]^+^ (Fig. 4b). Thirdly, the element composition was compared to those registered in the databases, and the metabolite was preliminarily identified as LysoPC (16:1). Lastly, mass fragmentation experiment was conducted to confirm the identification. The fragments of m/z 86, 104, 184, and 476 were observed in MS/MS spectrum (Fig. 4c), where m/z 86.0970 stands for [CH_2_CHN(CH_3_)3]^+^, m/z 104.1073 stands for [HOCH_2_CH_2_N (CH_3_)3]^+^, and 184.0732 stands for [H_2_O_3_PO-CH_2_CH_2_N(CH_3_)3]^+^. Both of them are typical fragments of PC, and moreover, 476.3133 stands for [M-H_2_O+H]^+^, further supporting the suggestion that this metabolite belongs to the class of LysoPC; all these data showed very good accordance with standard spectrum in Metlin databases (Fig. 4d). Based on all the inference, the biomarker was identified as LysoPC (16:1).

### The changes of the metabolic profile

**Fig. 5:**
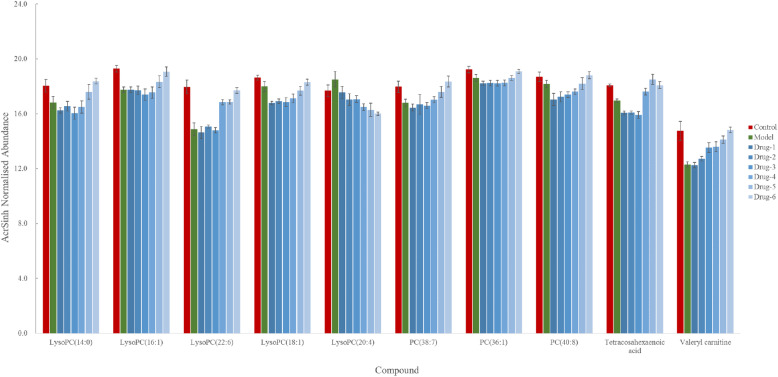
The relative intensity changes of the potential metabolites from different groups

Based on the protocol above, 11 potential biomarkers were identified. The change trend of potential biomarkers in different groups were shown in Fig. 5. The normalized abundance of metabolites were transformed by arcsinh function because their distributions were skewed. The arcsinh was incremental function and it could describe the trend of contents of metabolites in every group. The red column was control group, green column was model group, and the blue columns were drug group at different time. After injecting the prednisolone acetate solution, the contents of some metabolites (LysoPC (14:0), LysoPC (16:1), LysoPC (22:6), PC (38:7), PC (36:1), LysoPC (18:1), PC (40:8), tetracosahexaenoic acid, and valeryl carnitine) in model group were significantly lower than those in control group (*P* < 0.05). The contents of some metabolites (LysoPC (20:4)) of model group were significantly higher than those in control group (*P* < 0.05). And it was found that the relative intensity of most of these main metabolites in drug 5 weeks group and 6 weeks group were closer to normal control group, while drug 1 week group, 2 weeks group, and 3 weeks group were closer to model group.

### Heat map of the metabolites and metabolic pathway analysis

**Fig. 6:**
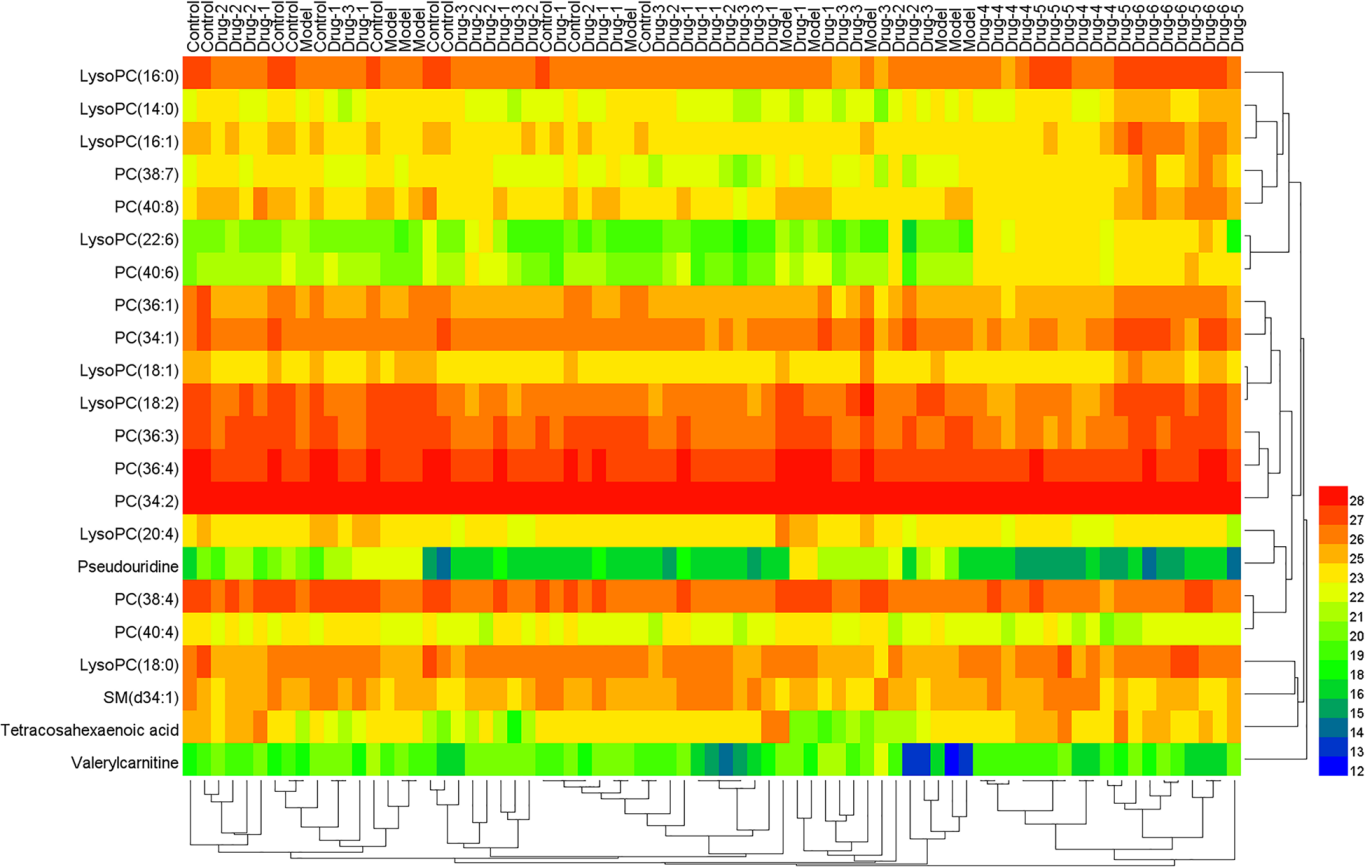
Heat map of the metabolites from different groups

According to the peak area of each metabolite of all groups, Heml 1.0.3.7 software packages were used to make a heat map in order to analyze these differential metabolites globally. The horizontal axis of the figure showed a dendrogram of the samples. The concentrations of different metabolites varied significantly in the different group. The vertical axis of the figure showed a dendrogram of the metabolite differences. The relative amount of metabolites in each sample could be visually discovered. Significant clustering and relevance of the metabolites was shown in the heat map (Fig. 6).

To explore the possible metabolic pathways after drug administration, we performed pathway analysis by metaboanalyst 3.0. Pathway impact plots were used to visualize the impact of altered metabolic pathways (Supplementary Fig. 2). The biological pathway analysis revealed six main metabolic pathways, including glycerophospholipid metabolism, linoleic acid metabolism, sphingolipid metabolism, alpha-linolenic acid metabolism, pyrimidine metabolism, and arachidonic acid metabolism. Among those, the *P* value of glycerophospholipid metabolism, linoleic acid metabolism, sphingolipid metabolism, and alpha-linolenic acid metabolism were less than 0.05, with the impact value 0.096, 0.077, 0.05, and 0.042, respectively, were filtered out as the most important metabolic pathways. The detail of pathways were listed in the Supplementary Table 1.

### Observation on histopathology and magnetic resonance imaging scan

**Fig. 7:**
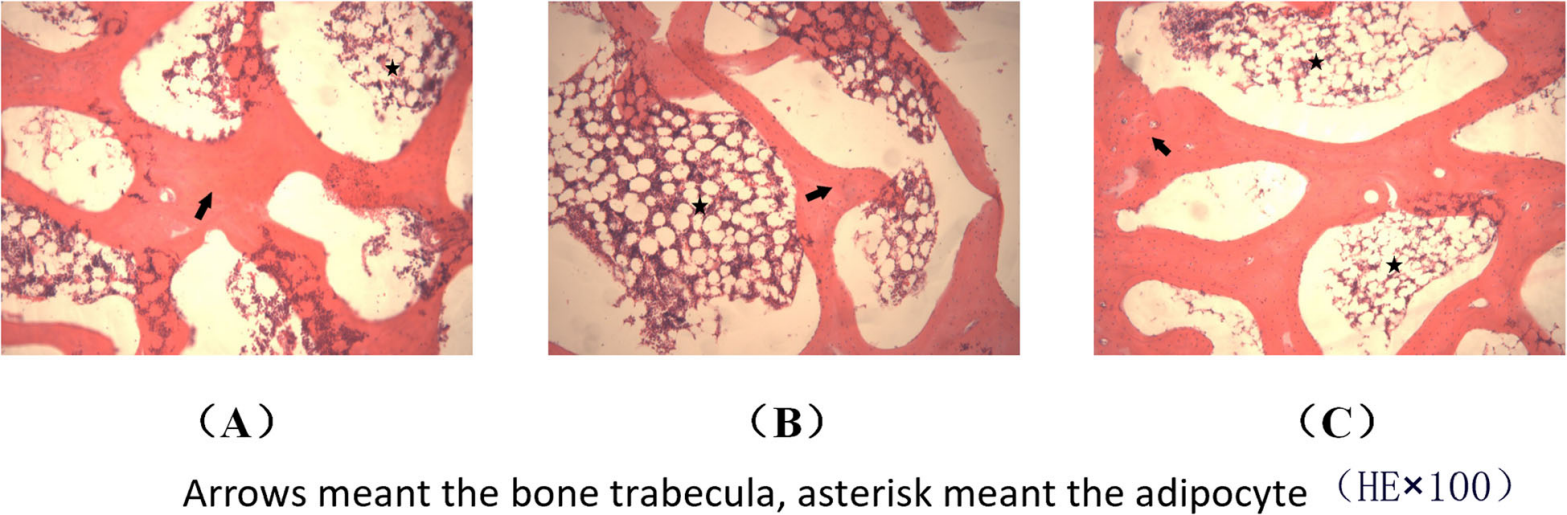
Results of HE staining of the femoral heads in three groups (× 100). **a** Control group, **b** model group, and **c** drug group

**Fig. 8:**
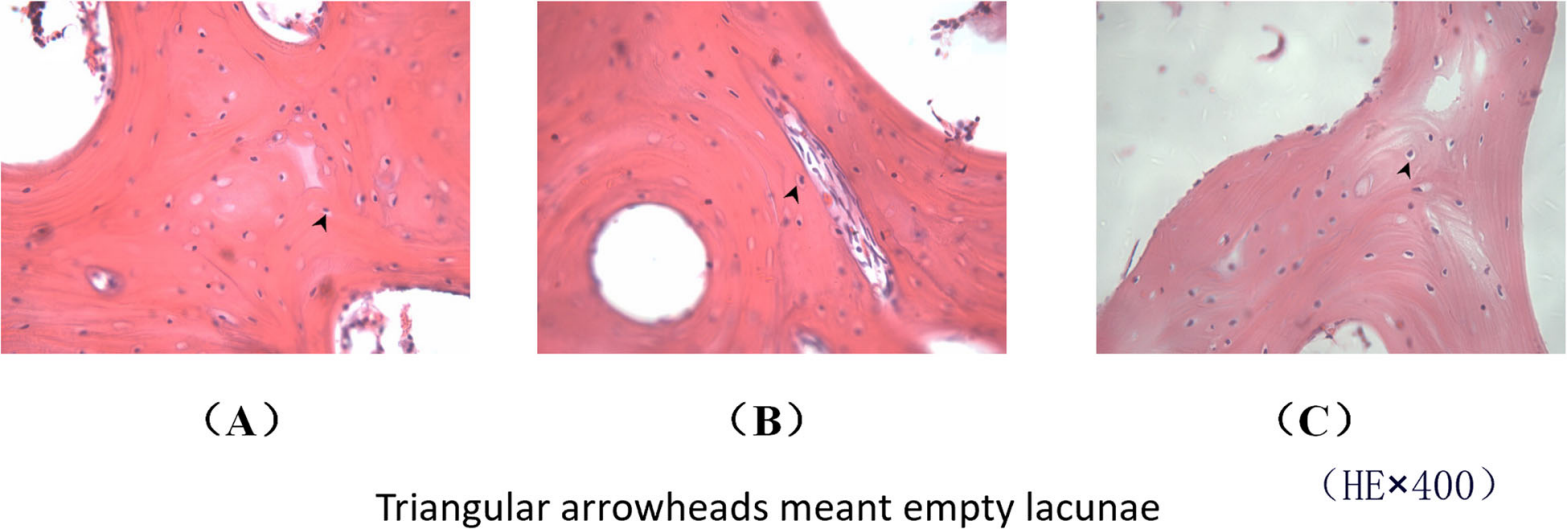
Results of HE staining of the femoral heads in three groups (× 400). **a** Control group, **b** model group, and **c** drug group

**Fig. 9:**
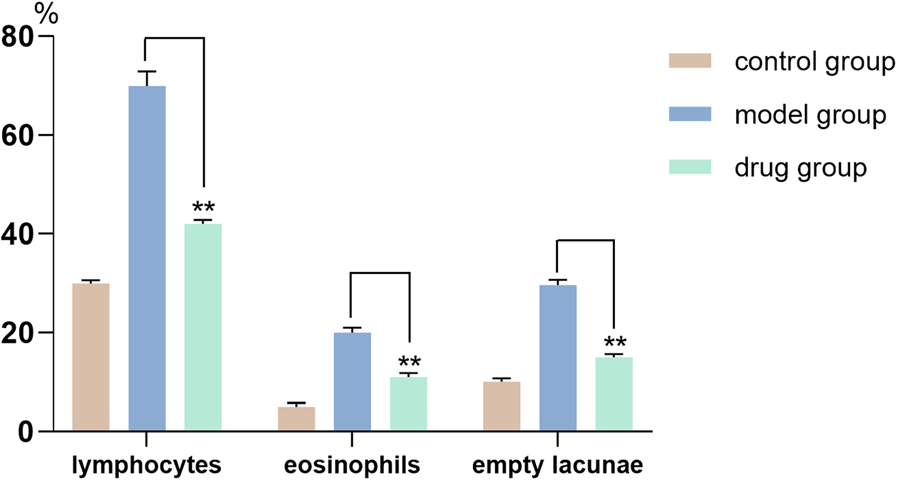
Quantitative analysis of histopathology on the sixth week

The changes of bone trabecula and adipocyte were shown in Fig. 7 and the changes of empty lacunae were shown in Fig. 8. The quantitative analysis of histopathology was shown in Fig. 9. In the control group, there were rich hematopoietic cells and relatively fewer lipocytes and eosnophils. The bone trabeculas were regularly arrayed, with complete structure, clearly visible osteocytes and a few empty lacunaes, and the empty lacunae rate is about (10.07 ± 0.72)% (Figs. 7a and 8a). In the model group, typical osteonecrosis was presented. Bone trabeculas turned thinner, ruptured, and irregularly arrayed. Many empty lacunaes were observed and the empty lacunae rate is (29.69 ± 1.05)%. There were significant differences between the two groups compared with the control group. The lipocytes were enlarged and there were rich lipocytes and eosinophils (Figs. 7b and 8b). In the drug group, there were a few lipocytes and eosinophils. The bone trabeculas irregularly arrayed, and only a few empty lacunaes were observed (Figs. 7c and 8c). Compared to the model group, lymphocytes and eosinophilic cells were significantly reduced in the drug group. After calculating the average number of cells in the field, the proportion of lymphocytes in the model group was 70%, which could be reduced to 41% after drug intervention, but still higher than that in the control group. Similarly, when counting eosinophils, the drug group reduced cell proliferation by almost half. Significant differences compared with model group were designated as **P < 0.05 (Fig. 9). Besides, the empty lacunae rate in drug group is (15.04 ± 0.65)%, which is significantly different compared with the model group.

**Fig. 10:**
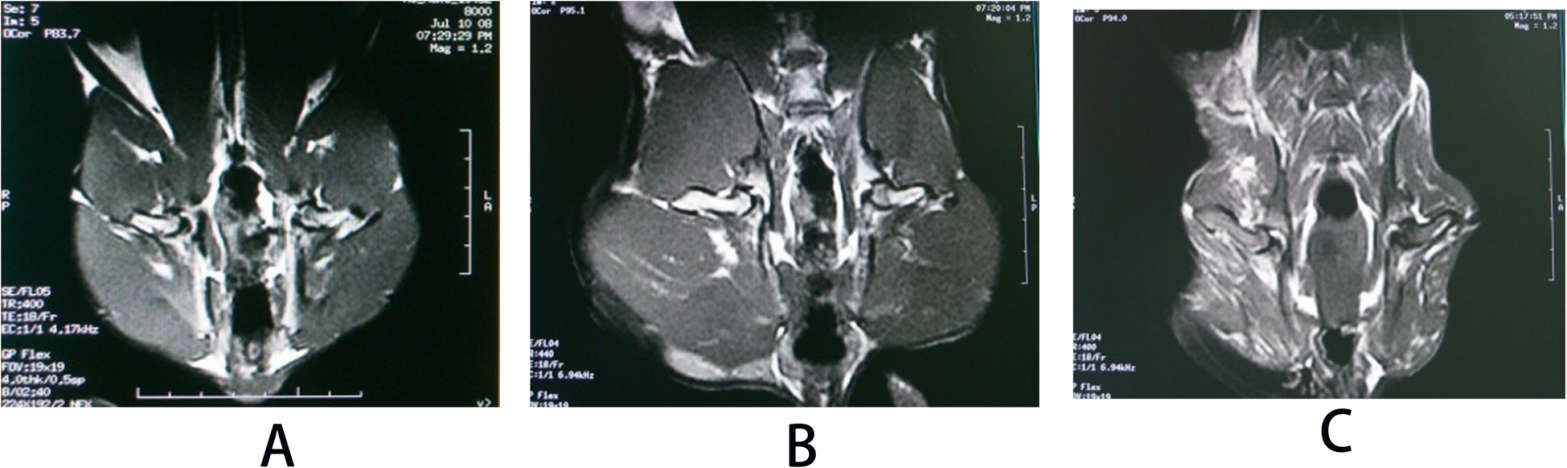
MRI (T2WI) of bilateral femoral heads of rabbits in three groups. **a** Control group, **b** model group, and **c** drug group

In the control group, the signal of femoral head was normal, and there was no abnormality inside and outside the joint soft tissue (Fig. 10a). In the model group, the bilateral femoral heads slightly got collapsed and flattened. The intensity of T_2_WI showed heterogeneous high-signal intensity. There were increasing hydrops in the right articular cavity and the gap of bilateral hip joints were uneven. The hydrops of bilateral spatium inter muscle are increased and there were rich hydrops in subcutaneous soft tissue (Fig. 10b). In the drug group, there were no apparent signals in bilateral femoral head and soft tissue muscle were noted. The gap of bilateral hip joints were equilate. There were a little hydrops in right articular cavity (Fig. 10c).

## Discussion

The use of statins in the treatment of steroid-induced necrosis of the femoral head has become popular in the past years [10, 16]. As a clinical medicine, the statins have been proven useful in preventing steroid-induced necrosis of the femoral head in clinical treatment [15]. However, the key metabolic pathways and potential biomarkers were not clear.

Metabolomics offered the opportunity to explore the effect of mechanism of lovastatin on the treatment of SANFH. PCA and PLS-DA model could provide an overview of the groups and OPLS-DA model was used to screen out the representative metabolites. PCA analysis showed an apparently cyclic trend. Good reproducibility of total ion chromatograms of the quality control group and ideal values of *R*^2^*Y* and *Q*^2^ could make sure the reliability of analysis. The identification is one of the crucial and difficult process of current metabolomics studies. Potential metabolites were identified based on accurate mass measurements via UHPLC-MS/MS, comparison with theoretical isotopic patterns, MS/MS fragment or comparison with HMDB database, Metlin database, lipid maps, and chemspider database. Through identification, 11 potential biomarkers were screened out, mainly containing phosphatidylcholine (PC), which were closely related to the SANFH [4, 27].

Many researchers have suggested the possible pathogenesis of steroid-induced necrosis of the femoral head, such as lipid metabolism disorder, intravascular coagulation, microvascular injury, intraosseous hypertension, osteocyte apoptosis, enlarged fat cells and fat embolism, and so on [2–4, 8, 9]. Glucocorticoids could not only stimulate but also regulate the process of adipogenesis occurring in the bone marrow stroma [13]. Indeed, the hypotheses had the close relationship with each other. According to the previous studies and this study, it could conclude that the glucocorticoid-induced necrosis of the femoral head were closely related to the lipid metabolism disorder, osteocyte apoptosis, and the hypertrophy and hyperplasia of the fat cells in the bone marrow. Lovastatin, as one of the lipid-lowering drug, could not only adjust the lipid metabolism but also decrease the adipogenesis in the bone marrow stroma [13]. Cui Q et al. showed that lovastatin inhibited steroid-induced fat-specific gene expression and counteracted the inhibitory effects of steroids on osteoblastic gene expression in vitro study [37]. Lovastatin may shunt uncommitted osteoprogenitor cells in marrow from the adipocytic pathway to the osteoblastic pathway by enhancing osteoblast genes (*PPARγ2* and *422aP*) expression and by inhibiting adipogenesis [38].

Our results showed that lovastatin might contribute to prevent and cure the SANFH. The cause of the steroid-induced osteonecrosis maybe multifactorial. There may be several possible mechanisms for this effect. Firstly, the pathways of glycerophospholipid metabolism, linoleic acid metabolism, sphingolipid metabolism, alpha-linolenic acid metabolism, and arachidonic acid metabolism belonged to the lipid metabolism. Researchers have reported that the statins could cure the SANFH and the possible mechanism was correcting the lipid metabolism disorder [15, 16]. Lipid metabolism was the core metabolism and was closely associated with several hypotheses [2–4, 8, 9]. Lovastatin, as one of the statins, was the lipid-lowering drug. Some researchers found that abnormal lipid metabolism is a strong risk factor, which may be the key pathomechanism of steroid-induced necrosis of femoral head [39]. Therefore, adjusting the lipid metabolism may be the important reason for lovastatin treatment of SANFH. Additionally, compared with control group, the contents of 7 potential biomarkers were lower in model group and they were phospholipids, namely LysoPC (14:0), LysoPC (16:1), LysoPC (22:6), PC (38:7), PC (36:1), LysoPC (18:1), and PC (40:8). For model group, the decreased phospholipid led to the increased permeability of cytomembrane, which occurred in the process of apoptosis. The apoptosis was closely related to the femoral head necrosis [40, 41]. After lovastatin administration group, the contents of these phospholipids increased. The degree of apoptosis may be alleviated. The lovastatin cured the SANFH which may have a relationship with alleviating the apoptosis. Moreover, lovastatin could prevent development of steroid-induced osteonecrosis in rabbits by inhibiting adipogenesis [13]. As the results of histopathology in this study, the lipocytes were enlarged and there were rich lipocytes and eosinophils in model group. In control group, there were rich hematopoietic cells and relatively fewer lipocytes and eosnophils. In drug group, there were a few lipocytes and eosinophils. The lipocytes were representative change in the experiment process. The steroids induced a significant enlargement of the adipocyte size and a fat accumulation in the bone marrow, which contributes to an intraosseous hypertension and a decreased blood flow [2–4]. The results of our study support the previous research findings that the use of lovastatin with SANFH could have a potential to preserve the bone mass by adjusting the lipid metabolism, inhibiting adipogenesis and delaying the osteocyte apoptosis to decrease the incidence of osteonecrosis, and it is meaningful for patients that need long-term or high-dose glucocorticoid therapy.

Glucocorticoid is widely used and has a significant effect. However, due to the side effects of glucocorticoid, the incidence of femoral head necrosis in patients requiring long-term or excessive use of glucocorticoid is increasing. Therefore, how to prevent SANFH has been widely a concern by the world medical community. Lipid metabolism disorders and bone cell apoptosis have been regarded as the important pathological basis of the pathogenesis of SANFH, and are currently the focus of research. This paper selected the lovastatin to intervene SANFH. Compared with other researches, the trends of femoral head histopathology and MRI were basically the same trend [37]. At the base of other researches, this study highlighted the phospholipid metabolism disorder by hormone and the development trend of femoral head necrosis under intervention effect of lovastatin. Therefore, there were limitations to study the pathogenesis and intervention mechanism of SANFH only from lipid metabolism disorder such as low-density lipoprotein, and total cholesterol and triglyceride. The phospholipid metabolism disorder may be the new pathway in intervention of SANFH.

Nevertheless, our research was novel, but there were some limitations. Firstly, the serum samples were collected from rabbits, which could provide the information of treatment for the use of lovastatin on SANFH. The samples from clinical patients needed further study. Moreover, the kinds of statins were not considered and we focused on the effect of lovastatin on SANFH. Additionally, this study gave the possible reason why lovastatin could prevent the SANFH. But the precise mechanism still requires a further in vivo study.

## Conclusion

In conclusion, untargeted metabolomics reveals that the significant changes were showed during treatment of on steroid-induced necrosis of the femoral head in rabbits after injected the lovastatin. In the process of trial, 11 potential metabolites changed and they mainly involved in six metabolic pathways. Combining the observation on histopathology and magnetic resonance imaging scan, we inferred that lovastatin could significantly decrease the incidence of osteonecrosis in the steroid-induced rabbits. The possible reasons were closely associated with adjusting the lipid metabolism, inhibiting adipogenesis, and delaying the osteocyte apoptosis.

## Supplementary information

**Additional file 1.**

**Additional file 2.**

**Additional file 3: Table 1.** The detail of pathways.

## Data Availability

All data generated or analyzed during this study are included in the manuscript.

## Abbreviations

SANFH: Steroid-induced avascular necrosis of the femoral head
UHPLC-MS/MS: Ultra-high performance liquid chromatography tandem mass spectrometry analysis
PCA: Principal component analysis
PLS-DA: Partial least squares-discriminate analysis
OPLS-DA: Orthogonal partial least squares-discriminant analysis
MRI: Magnetic resonance imaging
QC: Quality control
TIC: Total ion current
HCA: Hierarchical cluster analysis
HMDB: Human metabolome database
TEM: Transmission electron microscopy
PC: Phosphatidylcholine
