# Supplementary figures and images for: Untargeted metabolomics reveals the effect of lovastatin on steroid-induced necrosis of the femoral head in rabbits
Source: J Orthop Surg Res. 2020 Oct 28;15:497. doi: 10.1186/s13018-020-02026-5 (PMC7594276; doi:10.1186/s13018-020-02026-5)

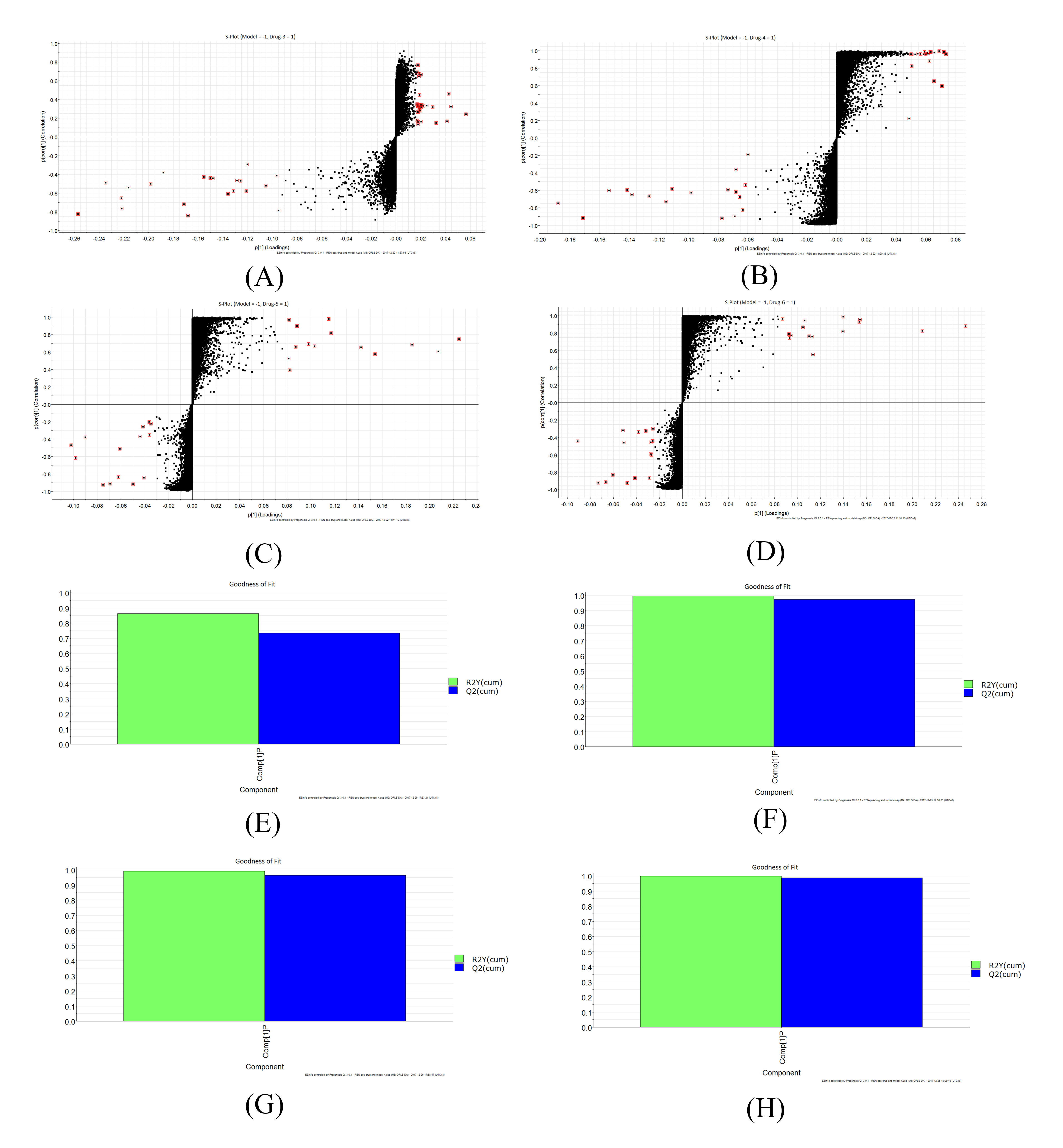

Supplement: Supplementary file 1 — Additional file 1. [file 13018_2020_2026_MOESM1_ESM.tif]

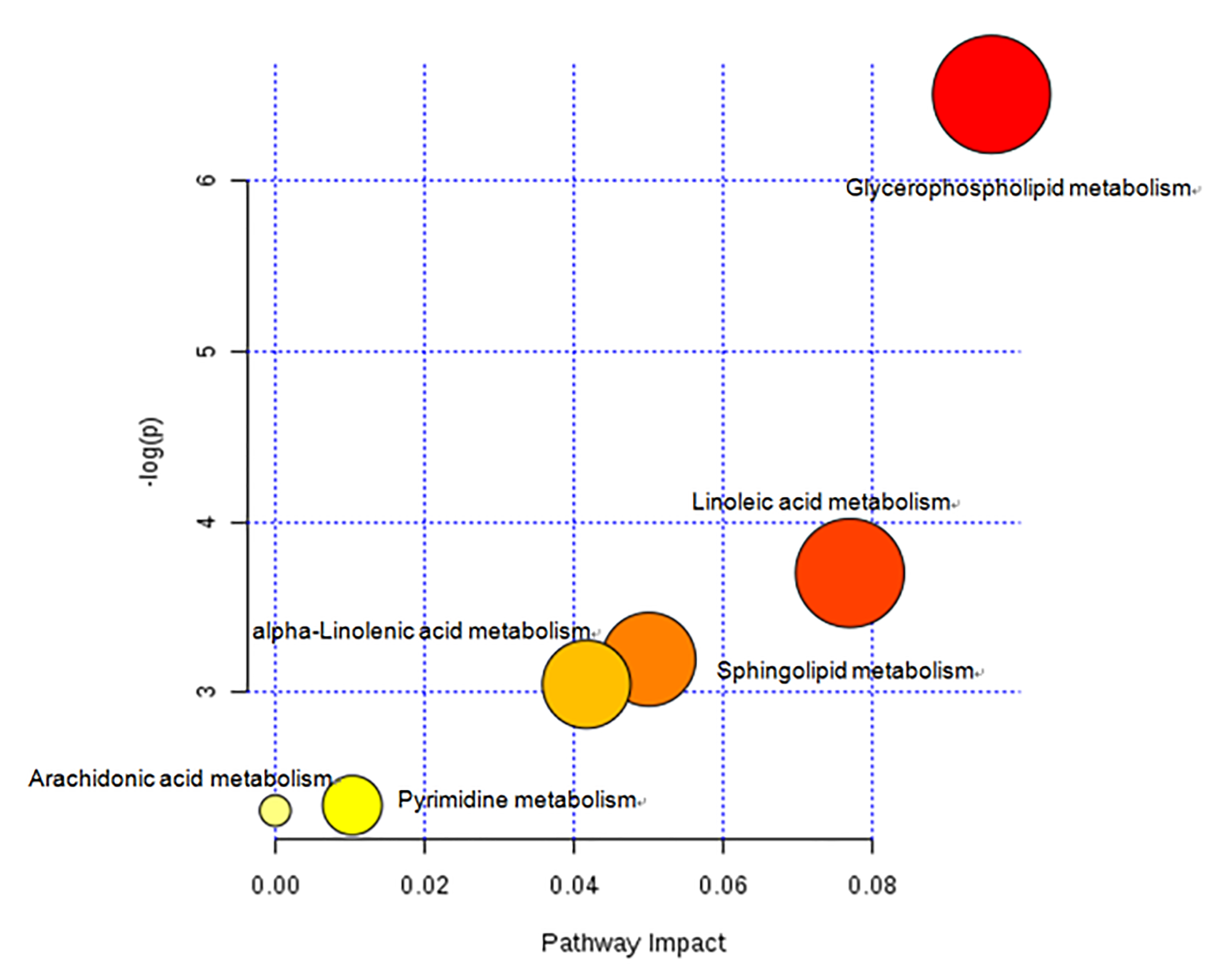

Supplement: Supplementary file 2 — Additional file 2. [file 13018_2020_2026_MOESM2_ESM.tif]
