# Supplementary material for: Untargeted metabolomics reveals the effect of lovastatin on steroid-induced necrosis of the femoral head in rabbits
Source: J Orthop Surg Res. 2020 Oct 28;15:497. doi: 10.1186/s13018-020-02026-5 (PMC7594276; doi:10.1186/s13018-020-02026-5)
Supplement: Supplementary file 3 — Additional file 3: Table 1. The detail of pathways. [file 13018_2020_2026_MOESM3_ESM.docx]

Table 1 The detail of pathways

| Pathway Name | p | -log(p) | Holm p | FDR | Impact |
| --- | --- | --- | --- | --- | --- |
| [Glycerophospholipid metabolism](http://www.metaboanalyst.ca/faces/Secure/pathway/ResultView.xhtml) | 0.002 | 6.500 | 0.120 | 0.120 | 0.096 |
| [Linoleic acid metabolism](http://www.metaboanalyst.ca/faces/Secure/pathway/ResultView.xhtml) | 0.025 | 3.701 | 1.000 | 0.947 | 0.077 |
| [Sphingolipid metabolism](http://www.metaboanalyst.ca/faces/Secure/pathway/ResultView.xhtml) | 0.041 | 3.196 | 1.000 | 0.947 | 0.050 |
| [alpha-Linolenic acid metabolism](http://www.metaboanalyst.ca/faces/Secure/pathway/ResultView.xhtml) | 0.047 | 3.050 | 1.000 | 0.947 | 0.042 |
| [Pyrimidine metabolism](http://www.metaboanalyst.ca/faces/Secure/pathway/ResultView.xhtml) | 0.096 | 2.342 | 1.000 | 1.000 | 0.010 |
| [Arachidonic acid metabolism](http://www.metaboanalyst.ca/faces/Secure/pathway/ResultView.xhtml) | 0.099 | 2.311 | 1.000 | 1.000 | 0.000 |
